# Modification of the Moxa

**Published:** 1847-07

**Authors:** 


*Modification of the Moxa.*—M. Guepratt proposes to use, in the place of cotton or amidon, paper which has been dipped into a solution of subacetate of lead, and afterwards dried; or he would prefer cotton so treated, to paper. This he tears in strips, and rolls into small rollers, which he makes to adhere at first, on the part to be treated, by a solution of gum arabic.—*Ibid.*

